# Coronary dominance among the Indian aborted fetal hearts

**DOI:** 10.6026/97320630018513

**Published:** 2022-06-30

**Authors:** Ashish Khokhariya, Sanjay Vikani, Bharat Gujar, Mayankkumar Javia, Ashok Nirvan

**Affiliations:** 1 Department of Anatomy, Banas Medical College & Research Institute, Palanpur, Gujarat, India; 2 Department of Anatomy, Shantabaa Medical College & General Hospital, Amreli, Gujarat, India; 3 Department of Anatomy, B J Medical College, Ahmedabad, Gujarat, India

**Keywords:** Right coronary artery, left coronary artery, posterior inter ventricular artery, coronary dominance, co-dominance

## Abstract

Increasing incidences of myocardial infarction and decreasing age at which they are occurring has forced many researchers to do in depth study pertaining to the anatomical variations in the vascular pattern of heart. Coronary dominancy of the heart will determine whether the territory of the heart supplied by the posterior inter ventricular artery will receive blood from right coronary artery or left coronary artery or both. Present study was conducted to explore the variations in the coronary dominant pattern in the aborted human fetal hearts. Right and left coronary arteries in 30 aborted human fetal hearts were thoroughly dissected from their commencement from the corresponding aortic sinus till their termination. The coronary dominance was determined on the basis of origin of posterior inter ventricular artery. We found 60% cases of right coronary dominance, 36.66% cases of left coronary dominance and 3.33% cases of balanced coronary dominance/ coronary co-dominance. Data shows variations in the vascular dominancy pattern of heart can be critically important for the cardiac surgeons, cardiologists as well as interventional radiologists while performing investigational or operative procedures.

## Background:

The human heart is a hollow, somewhat conical muscular organ, situated in middle mediastinum and covered by pericardium. It pumps blood to various part of body to meet their nutritive requirement. The Latin name for the heart is "cor" from which we have the adjective coronary. It consist of four chamber right atrium, right ventricle, Left atrium, left ventricle and these chambers are separated by inter atrial & inter ventricular septum. [1] Coronary arteries are the enlarged vasa vasorum and supply the myocardium & epicardium of heart. The blood flow is maximum during diastole & minimum in systole. There are right and left coronary arteries. There is no definitive demarcation between the areas of distribution of two coronary arteries. In majority of cases the area of distribution of left coronary artery is larger than that of right one. [2,3] These are the first branches of the aorta. They arise from the ascending aorta immediately above the aortic valve and initially pass around the opposite sides of the pulmonary trunk. The right coronary artery arises from the right aortic sinus of the ascending aorta. The left coronary artery, which is usually larger than the right coronary artery, supplies the major part of the heart. It arises from the left aortic sinus of the ascending aorta. Coronary arteries are functional end arteries, and if they are blocked by any disease, the cardiac muscle normally supplied by those arteries will receive insufficient blood and undergo necrosis. Blockage of a large coronary artery can cause severe cardiac arrest, which may lead to sudden death of the patient. [4,5] Mostly the right coronary artery gives posterior inter ventricular branch. Such hearts are right dominant. In about 10-30% of heart, the right coronary artery is rather small and is not able to give the posterior inter ventricular branch. In these cases the circumflex artery, the continuation of left coronary artery, provides the posterior inter ventricular branch as well as branch to the AV node. Here the left coronary artery is dominant and sometimes refers as 'the widow maker' artery, as occlusion of the main stem of the same usually results in death of a person. Such cases are called left coronary dominant. Thus the coronary artery which gives the posterior inter ventricular branch will determine the coronary dominance of the heart. [6,7] The cardiovascular system starts developing in the middle of the third week of the gestational age, when the embryo is no longer able to satisfy its nutritional requirements by diffusion alone. Once cells establish the primary heart field, they are induced by the underlying pharyngeal endoderm to form cardiac myoblasts and blood islands that will form blood cells and vessels by the process of vasculogenesis. With time, the islands unite and form a horseshoe-shaped endothelial-lined tube surrounded by myoblasts. This region is known as the cardiogenic region; the intra embryonic (primitive body) cavity over it later develops into the pericardial cavity. In addition to the cardiogenic region, other blood islands appear bilaterally, parallel, and close to the midline of the embryonic shield. These islands form a pair of longitudinal vessels, the dorsal aorta. [8] The mesenchymal cells in cardiogenic area condensed to form two angioblastic cords, which later canalized to form two endothelial heart tubes in 3rd week of intrauterine life. These tubes fuse with each other in cranio-caudal direction to form single primitive heart tube. The caudal ends of the heart tubes don’t fuse and remains bifurcated. The heart tube shows five dilatations from cranio-caudally; truncus arteriosus, bulbus cordis, primitive ventricle, primitive atrium and sinus venosus. Truncus arteriosus is the arterial end which is continuous above with the aortic sac, while the sinus venosus is the venous end of the developing heart tube. [9] Therefore, it is of interest to explore the variations in the coronary dominant pattern in the aborted human fetal heart in the form of right coronary dominant, left coronary dominant, balanced coronary dominant/ coronary co-dominant.

## Materials and methods:

Present study was conducted in the Department of Anatomy, B J Medical College, Ahmadabad, Gujarat, India from 2015 to 2019 after taking due permission from the institutional ethics committee. 30 aborted human fetuses (13 male and 17 female fetuses), having gestational age between 14 to 40 weeks, were collected from the Department of Obstetrics and Gynecology, B J Medical College, Ahmadabad, Gujarat, India. Written consent in the regional language was taken from the parents to use the aborted fetuses for educational and research purpose. In the present study, fetuses below the gestational age of 14 weeks; grossly macerated fetuses; parents not giving written consent; unknown obstetric history; medico-legal cases; any visible gross deformity in the aborted fetuses were the exclusion criteria, while fetal gestational age between 14 to 40 weeks; known obstetric history; written consent given by the parents of aborted fetus were the inclusion criteria. Gestational age of fetus was determined with the help of obstetric history, date of last menstruation period and Ultra Sonography findings of the mother. All 30 fetuses were embalmed by injecting 10% formalin into the umbilical vessels. Thoracic cavity of the fetuses was opened by using anterior midline incision on the thorax and heart was removed by opening the pericardial sac. In each heart, right and left coronary artery were carefully dissected from their commencement from corresponding aortic sinus till their termination by using pointed forceps and needle. The coronary dominance was determined on the basis of origin of posterior inter ventricular artery. If the posterior inter ventricular artery arises from right coronary artery it is called right coronary dominance. If the posterior inter ventricular artery arises from the left coronary artery it is called as left coronary dominance and if the posterior inter ventricular artery arises from both the coronary arteries than it is called as coronary co-dominance (balanced dominance) (Figure 1 - see PDF). After complete study of coronary artery, photographs of each specimen were taken; results were recorded and analyzed statistically.

## Results:

Origin, course & branching pattern of coronary arteries were inspected carefully in each specimen. The coronary dominance was determined on the basis of origin of posterior inter ventricular artery. Out of total 30 specimens, 18 specimens (60 %) were of right coronary dominance and 11 specimens (36.66 %) were of left coronary dominance. In 1 out of total 30 specimens (3.33 %), the posterior inter ventricular artery arises from both right as well as left coronary arteries which is called balanced coronary dominance/ coronary co-dominance (Table 1; Figure 2 - see PDF).

## Discussion:

Most of the studies of coronary dominance available in the literature have been done in adult human heart (Table 2 - see PDF). The only study about the coronary dominance in the aborted human fetal heart was carried out by Jyothirmayi K et al [10] in 2014 (Table 2 - see PDF) and Yumnam B et al [11] in 2015 (Table 2 - see PDF). Jyothirmayi K et al found 56% (28 out of 50) cases of right coronary dominance, 38% (19 out of 50) cases of left coronary dominance and 6% (3 out of 50) cases of balanced coronary dominance/ coronary co-dominance. Yumnam B et al found 70% (21 out of 30) cases of right coronary dominance, 20% (6 out of 30) cases of left coronary dominance and 10% (3 out of 30) cases of balanced coronary dominance/ coronary co-dominance. Schelesinger et al (Table 2 - see PDF) found 48% cases of right coronary dominance, 18% cases of left coronary dominance and 34% cases of balanced coronary dominance/ coronary co-dominance cases in adult human heart. [12] Aricatt DP et (Table 2 - see PDF) al found 85.5% cases of right coronary dominance, 9.7% cases of left coronary dominance and 4.8% cases of balanced coronary dominance/ coronary co-dominance cases in adult human heart. [13] James et al (Table 2 - see PDF) found 90% cases of right coronary dominance and 10% cases of left coronary dominance in adult heart. They did not found any cases of balanced coronary dominance/ coronary co-dominance. [14] In the present study, we found 60% (18 out of 30) cases of right coronary dominance, 36.66% (11 out of 30) cases of left coronary dominance and 3.33% (1 out of 30) cases of balanced coronary dominance/ coronary co-dominance in the aborted human fetal heart. Cavalcanti et al (Table 2 - see PDF) found 69.09% cases of right coronary dominance, 11.82% cases of left coronary dominance and 19.09% cases of balanced coronary dominance/ coronary co-dominance cases in adult human heart. [15] Ravi V et al (Table 2 - see PDF) found 83.3% cases of right coronary dominance and 13.3% cases of left coronary dominance and 3.3% cases of balanced coronary dominance/ coronary co-dominance cases in total 30 adult human cadaveric hearts they studied. [16] Lekshmy Vijay VG et al (Table 2 - see PDF) found 84% cases of right coronary dominance, 16% cases of left coronary dominance out of total 112 adult human cadaveric hearts they studied. They didn’t find any case of balanced coronary dominance/ coronary co-dominance. [17]

In left coronary dominant heart, the whole of the left ventricle, some part of right ventricle and left atrium as well as inter ventricular septum will receive blood from the left coronary artery. In right coronary dominant heart, the posterior inter ventricular artery will supply some of these areas, reducing the area supplied by the left coronary artery. This clearly states that the lesions of left anterior descending artery are more severe in left coronary dominant heart as compared to the right coronary dominant heart. Mostly the atrio ventricular node is supplied by the branches of right coronary artery. So an inferior wall infarct caused by the occlusion of right coronary artery will have higher risk of development of atrio ventricular nodal block. [18,19] In persons having coronary co-dominant heart, two posterior inter ventricular arteries arise each from the left coronary artery and right coronary artery. In case of obstruction of any one coronary artery, the posterior inter ventricular septal area will receive blood from the other coronary artery and the area affected due to deprivation of blood supply will be minimal. [20] After applying the multivariate conditional logistic regression analysis on the results found, Wang L et al stated that the right coronary dominance was closely related to the occurrence of acute inferior myocardial infarction. [21] With increasing age the prevalence of left coronary dominant and co-dominant coronary system decreases. This may be due to higher risk of mortality in cases of left dominant heart as compared to right. [22, 23]

## Conclusion:

Data shows the variations in the dominance pattern of coronary artery in aborted human fetal heart. Enhanced understanding of vascular dominance pattern of heart will be helpful to the cardiologists; cardiac surgeons as well as interventional radiologists for better success ratio in various investigational and operative procedures related to the coronary artery. Further these data can be utilized for the development of various preventive programs targeting the cardiac care.
